# Defining Cell-Surface Antigenic Markers for Mouse T and B Cells

**DOI:** 10.3389/fimmu.2014.00559

**Published:** 2014-11-20

**Authors:** Martin C. Raff

**Affiliations:** ^1^MRC Laboratory for Molecular Cell Biology, University College London, London, UK

**Keywords:** T cells, B cells, cell-surface markers, thy1, Ig, carrier effect, pre-B cells

I began my scientific career in October 1968 at the National Institute for Medical Research (NIMR) in London. I was 30-years old, having just finished my training in clinical neurology in Boston, and I had come to work with Avrion (Av) Mitchison. I had much to learn, as I had done no basic research and knew very little about immunology.

It was an exciting time – both in immunology and at the NIMR. There was increasing evidence that there were two types of lymphocytes, T and B cells, responsible for adaptive immune responses but there were no good ways to distinguish or separate them. Av had recently heard the Boston immunologist Arnold Reif describe a mouse isoantigen called theta (θ), which was present in the brain and on the surface of thymocytes (1, 2). Av wondered if θ might be present on T but not B cells, in which case it could serve as a useful cell-surface marker for mouse T cells. He gave me the relevant Reif papers, an aliquot of a mouse anti-θ antiserum that he had begun making, and set me free.

In a commentary written in 2008 for the Pillars of Immunology series in the *Journal of Immunology* (3), the Seattle immunologist Pamela Fink colorfully described what happened next. Here, I give an abbreviated account of this history but broaden it to include the fortuitous finding that immunoglobulin (Ig) can serve as a cell-surface marker for B cells.

To detect θ on mouse lymphocytes, I first used an antibody- and complement-dependent ^51^chromium-release cytotoxicity assay, which I learned from my lab-mate Marion Ruskowicz (4). I found that the antiserum and complement killed essentially all thymus lymphocytes but only a subset of lymph node and spleen lymphocytes. To test whether the θ-positive lymphocytes in lymph node and spleen were T cells, I analyzed cells from pathogen-free mice that had been treated since birth with a rabbit antiserum made against mouse thymocytes (5) and were therefore T-cell-depleted; Sandra Nehlson, a Ph.D. student with Peter Medawar who worked across the hall, generously provided these mice. I found that the spleen and lymph nodes of the mice contained normal numbers of θ-negative lymphocytes but greatly reduced numbers of θ-positive lymphocytes, strongly suggesting that Av’s hunch was right – θ is present on T but not B cells (6). Schlesinger and Yron independently published very similar findings around the same time (7); unfairly, their paper received far less attention, probably because its title lacked the punch line of their findings. Later, in collaboration with Henry Wortis, who worked next door, we confirmed these findings in other T-cell-deficient mice, including congenitally athymic nude mice (8).

I then tested the functional properties of θ-positive spleen cells by analyzing the cells involved in an adoptive cell-transfer system that Av had developed to study the cooperation between two populations of spleen cells – one from mice immunized with a hapten (NIP) coupled to a carrier protein (chicken γ-globulin, CGG) and another from mice immunized with an uncoupled, second carrier protein (bovine serum albumin, BSA). He had shown that, when both cell populations, but not either one alone, are transferred into a sub-lethally irradiated mouse, the recipient mouse produces large amounts of anti-NIP antibodies in the blood when immunized with NIP-BSA but not with NIP-CGG – an example of the so-called carrier effect (9). In my experiments, before transferring the cells, I treated one or other population with anti-θ antibodies and complement to kill the T cells, using normal mouse serum plus complement as a control. In this way, I could show that the relevant cells in the BSA-immunized population were T cells, whereas the relevant cells in the NIP-CGG-immunized population, which produced the anti-NIP antibodies (9), were not (10). This experiment provided direct evidence that T cells recognizing antigenic determinants on a protein can help B cells make antibodies against different antigenic determinants on the same protein (11). It also established the value of antibodies that recognize cell-type-specific surface antigens, which rapidly became standard tools in immunology and, later, in various other branches of biology.

Remarkably, Av declined to put his name on these two *Nature* papers (6, 10), even though the projects were his idea and he had begun to produce anti-θ antibodies before I arrived in London. This exceptional generosity had an enormous influence on my career. Theta (now called Thy-1) rapidly became a standard marker for mouse T cells, and the two single-author *Nature* papers gave me immediate international recognition, after only 2 years doing basic science. Nonetheless, if I had known then what I know now, I would have insisted that Av’s name was on the papers, to indicate his crucial contributions to the work. Av always did his own experiments and made many landmark contributions to immunology; because he usually allowed his students and postdoctoral fellows to publish on their own, however, his actual contributions are far greater than are documented in the literature.

To visualize θ directly on the surface of living T cells, I turned from cytotoxicity assays to immunofluorescence experiments. In these experiments, I visualized the bound mouse anti-θ antibodies using fluorescent rabbit anti-mouse-Ig antibodies. A surprise came from control experiments in which I omitted the anti-θ antibodies and found that the fluorescent anti-Ig antibodies on their own labeled a substantial proportion of lymphocytes in cell suspensions prepared from various peripheral lymphoid organs, although not in suspensions of thymocytes. Roger Taylor, working across the hall with Michel Sternberg, had independently obtained similar results using radiolabeled anti-mouse-Ig antibodies, and we published our findings together (12). Although a number of immunologists, including Av, had suspected that the antigen receptors on lymphocytes might be Ig proteins, ours was one of the first direct demonstrations of Ig molecules on the surface of lymphocytes.

The finding of Ig on some peripheral lymphocytes but not others raised the question of which class of lymphocyte expressed the cell-surface Ig. To find out, I studied lymphocytes from normal mice and from various T-cell-depleted mice, labeling the cells with anti-mouse-Ig antibodies, with or without first labeling them with mouse anti-θ antibodies. The results were unambiguous: the Ig-positive cells were θ-negative, implying that they were B cells, whereas the θ-positive T cells were Ig-negative (13). (The analysis was greatly helped by the fact that the Ig was distributed in a cap at one pole of the B cells, whereas θ was distributed as a ring on the T cells; later, Stefanello de Petris and I, and Roger Taylor and Phillip Duffus independently, showed that the binding of the anti-Ig antibodies induces the B cells to actively redistribute their surface Ig molecules into a cap (14) – but that is another story.) The *Journal of Experimental Medicine* rejected my paper as not being sufficiently interesting, and it was published in *Immunology*, a low impact journal. Despite this (and its unhelpful title), it became a Citation Classic (15), which taught me an important lesson: it is what you publish rather than where you publish it that matters most. The finding of Ig on the surface of B cells but not T cells led to a prolonged and frustrating search by many laboratories for the antigen receptors on T cells, which were only identified as distinct Ig-like proteins years later, after a number of false leads (16).

Cell-surface Ig became a standard marker for B cells in all vertebrates. When I moved with Av to University College London, for example, John Owen and I collaborated with Max Cooper (who was on sabbatical from the University of Alabama) and used anti-Ig antibodies and explant cultures to study the development of B cells. We showed that mouse B cells develop in the fetal liver and adult bone marrow (17), rather than in gut-associated lymphoid tissues as had been proposed by Max and others. We later used Max’s class-specific anti-Ig antibodies to demonstrate that the B cells arise from pre-B cells, which have already begun to make IgM heavy chains (18).

Remarkably, these first few years in science were the most productive in my research career. This early success was largely the result of good luck: I was at the right place at the right time, with a generous and inspiring mentor. And it was why I became a scientist rather than a practicing neurologist.

## Conflict of Interest Statement

The author declares that the research was conducted in the absence of any commercial or financial relationships that could be construed as a potential conflict of interest.
